# The Potential Role of Basophils in Urticaria

**DOI:** 10.3389/fimmu.2022.883692

**Published:** 2022-05-17

**Authors:** Riko Takimoto-Ito, Ni Ma, Izumi Kishimoto, Kenji Kabashima, Naotomo Kambe

**Affiliations:** ^1^ Department of Dermatology, Kyoto University Graduate School of Medicine, Kyoto, Japan; ^2^ Department of Dermatology, Kansai Medical University, Hirakata, Japan

**Keywords:** basophil, urticaria, mast cell, IgE, omalizumab

## Abstract

Urticaria is a symptom of acute skin allergies that is not clearly understood, but mast cell histamine is hypothesized to cause swelling and itching. Omalizumab, an anti-human IgE antibody that traps IgE and prevents its binding to high-affinity IgE receptors, is effective in treating urticaria. We recently experienced a case of urticaria refractory to antihistamine therapy in which the peripheral-blood basophil count responded to omalizumab therapy and its withdrawal. Furthermore, the peripheral-blood basophils showed an unexpected increase in the expression of a cell surface activation marker. This phenomenon has been reported by other analyses of basophil and mast cell dynamics during omalizumab treatment. Here, we analyze these observations and formulate a hypothesis for the role of basophils in urticaria. Specifically, that activated basophils migrate to the local skin area, lowering peripheral-blood counts, omalizumab therapy alters basophilic activity and causes their stay in the peripheral blood. We hope that our analysis will focus urticaria research on basophils and reveal new aspects of its pathogenesis.

## Introduction

Urticaria is a common skin disease characterized by itchy, red, raised bumps or hives. It is generally classified based on its duration, acute (≦ 6 weeks) or chronic (>6 weeks), and the relevance of eliciting factors, spontaneous (no specific eliciting factor involved) or inducible (specific eliciting factor involved) (1). Based on these classifications, chronic spontaneous urticaria (CSU) is diagnosed based a duration of over 6 weeks and the absence of identifiable eliciting factors.

The etiology and pathogenesis of CSU remain largely unexplored. However, there is a consensus that cutaneous mast cells play a major role by releasing histamine, which acts on blood vessels and nerves to cause swelling and provoke itching, respectively. Antihistamine is the standard treatment for urticaria and antihistamine-resistant patients are well-controlled with omalizumab, an anti-human IgE antibody, which reconfirms that IgE is important in the pathology of urticaria, even in CSU with unknown triggers.

## Peripheral Blood Basophil Counts and the Disease Activity of CSU

We recently reported a case with a 6-year history of severe CSU, whose peripheral basophil count was persistently low (almost zero cells per µL) for more than 1 year (2). Upon urticaria resolution by omalizumab administration, we noticed that the peripheral basophil count had increased. When the patient discontinued omalizumab, the peripheral blood basophil count again dropped to zero and urticaria recurred. Restarting omalizumab rescued the peripheral blood basophil count and improved the skin rash. Before our observation, this inverse correlation with basophil count and urticarial activity has been confirmed by clinical trials of omalizumab (3) in which they quantified peripheral-blood basophils using blood histamine levels and flow cytometry. The study authors found that both parameters increased with omalizumab treatment and clinical CSU improvement.

The contribution of basophils to allergic reactions, acquired immunity, and autoimmune diseases is better understood (4, 5), though it was long ignored or confused with that of tissue-resident mast cells. This may be because basophils account for less than 1% of granulocytes in the peripheral blood and are phenotypically similar to mast cells. Both cell types have basophilic granules in their cytoplasm, high-affinity IgE receptors (FcϵRI) on their surface, and release histamine and other chemical mediators.

The fact that basophils express FcϵRI and release histamine, together with observation that the basophils in peripheral blood inversely correlate with urticarial rash severity, suggests that basophils play a role in CSU. Indeed, as omalizumab has become more frequently used in the treatment of CSU refractory to antihistamine therapy, a number of reports have examined potential predictors of its efficacy (6–10). Many studies have demonstrated that low total IgE levels are an essential marker for discriminating the efficacy of omalizumab. Fok et al. (9) found that low IgE levels in CSU patients did not respond to omalizumab or had only a weak effect, but there was no evidence to suggest which marker was effective. Aghdam et al. (10) evaluated whole blood leukocytes responses in CSU patients treated with omalizumab to elucidate the effect of omalizumab on different FcϵRI-bearing leukocytes, only percentage of basophil increased after omalizumab treatment, but other white blood cells remain stable. As for baseline characteristics of CSU patients, Johal et al. (6) reported higher symptom scores and slower symptom improvement with omalizumab treatment in those with decreased peripheral blood basophils than in those without, which is consistent with previous studies focusing on basopenia as a marker of disease severity (11). Rijavec et al. (7) reported that a very low number of absolute basophil count in the circulation (1.7 basophils/µL) is predictive of a poor response to omalizumab. Furthermore, low circulating basophils count significantly correlated with very low densities of FcϵRI and IgE on basophils. They also confirmed the basopenia using the low expression of basophil-related genes in the whole-blood genes, including *FCER1A* that codes α-chain of FcϵRI, *CPA3* that codes carboxypeptidase A3, and *HDC* that involves histamine synthesis.

The phenomenon that basophils are inversely correlated with CSU severity is not specific to omalizumab treatment, as it was also observed in CSU patients treated with antihistamines (12). This report measured symptom severity using the daily baseline urticaria activity score; the more severe the symptoms, the lower the peripheral blood basophil count. Thus, the relationship between peripheral-blood basophils and urticaria severity is not treatment-method dependent.

In clinical practice, basophils account for a very small percentage of peripheral blood cells, which makes it difficult to define an expected value. However, basophil fluctuations can indicate urticaria activity, at least on a patient-by-patient basis.

## A Basophil Activation Marker Increased After the Initiation of Omalizumab

Not only did omalizumab treatment affect peripheral-blood basophil levels (2), it also increased their surface expression of CD203c, which reflects the activation state of basophils (13). It seems a paradoxical phenomenon where peripheral circulating basophils appear to express more CD203c after successful omalizumab treatment. Of course, it is important to consider that CD203c expression is not equivalent to CD63 expression and is not a specific marker for degranulation. Its expression can be driven by IL-3 at least *in vitro* (14), besides IgE-FcϵRI cross-linking (15). Therefore, some reports suggest that CD203c expression is not a suitable index for assessing basophil activation in patients with CSU (14).

However, omalizumab has also been reported to enhance sensitivity of human basophils to IgE-mediated stimulation (16–21). Basophils collected from the patients with the sensitivity to cat allergens showed 3- to 125-fold more sensitive to antigen-driven secretion after the treatment with omalizumab (17). Nevertheless, the lack of change in responsiveness to non-IgE-mediated stimuli, e.g., to N-formyl-methionyl-leucyl-phenylalanine, may be worth considering (18).

**Figure 1:**
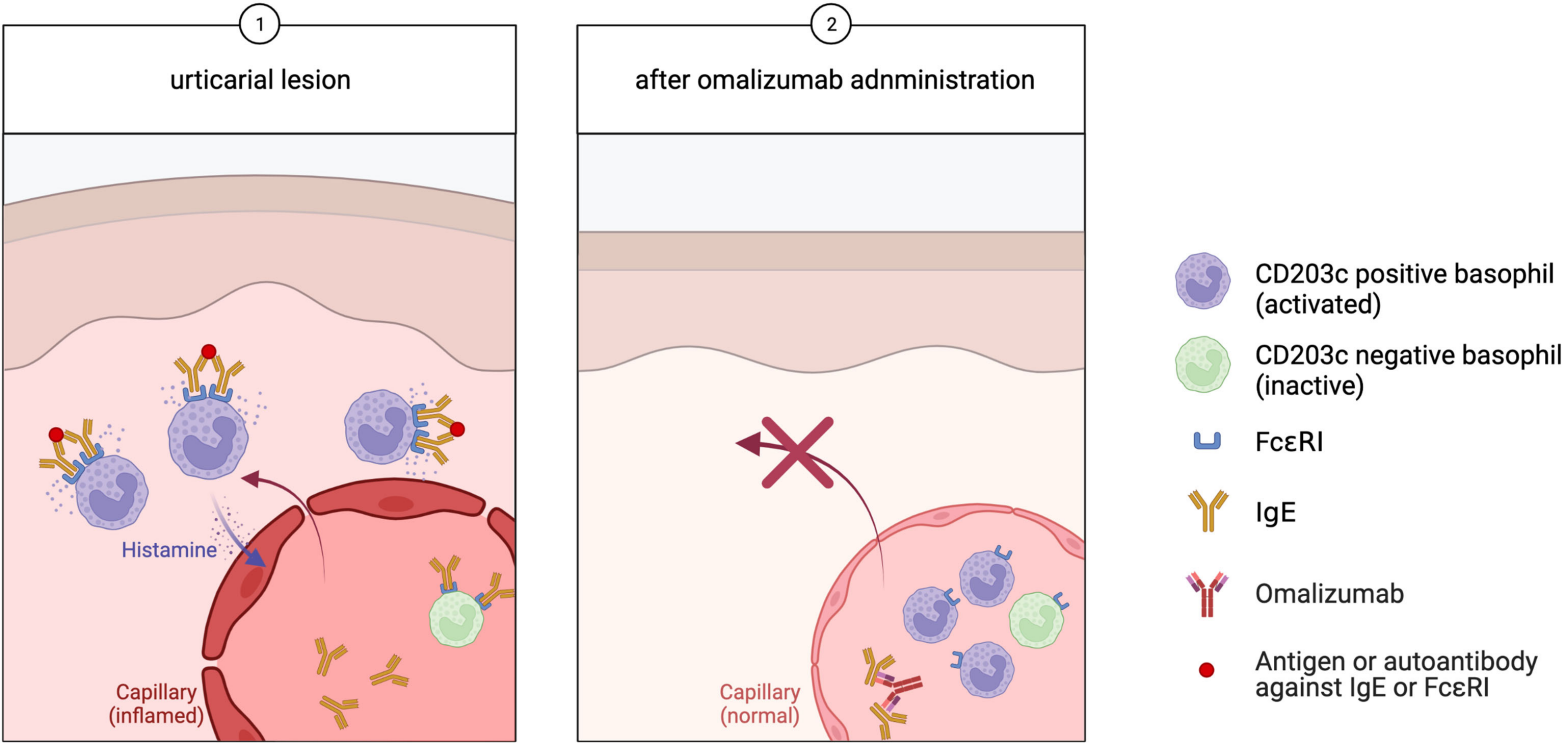
In urticaria, (Step 1) activated basophils migrate to the skin lesions, while inactive basophils remain in the peripheral blood. (Step 2) Omalizumab binds free IgE, which prevents further basophilic activity and a corresponding decrease in FcϵRI expression. Activated CD203c-positive basophils stay in the peripheral blood, leading to an increase of the peripheral blood basophil population.

To explain this, we hypothesized that activated basophils migrate locally to the skin while patients experience CSU symptoms, and inactive or immature basophils unable to show sufficient activity in response to IgE-mediated stimulation remain in the peripheral blood (**Figure 1**, step 1). Once the CSU symptoms are relieved, the basophils with the ability to become fully activated in response to stimuli stay in the peripheral blood, increasing the frequency of peripheral blood basophils that express cell-surface CD203c.

## Basophil Infiltration of CSU Lesions

The BB1 antibody against human basophils has detected basophil infiltration into skin lesions (22–24). In CSU patients, the number of mast cells in autologous serum-induced wheals remains constant from about 30 minutes to 48 hours after development. However, the number of basophils was high at baseline compared to healthy skin and further increased by about 30 minutes after onset (23). Moreover, the immunohistochemistry analysis of autologous wheals revealed overlapping trends of IL-4 (possibly expressed by activated basophils) and the chemokine receptor CCR3, which are primarily associated with type 2 immune responses. The presence of these molecules peaked at 30 minutes, then began to decline, which suggests that these recruiting mechanisms might attract basophils early in the reaction (23).

The relationship between blood and tissue basophil counts has not been studied in detail. However, given the finding that blood basopenia is frequently observed in CSU patients (25), these reports of the identification of basophils at sites of inflammatory lesions suggest that the decrease in basophils is probably the result of migration of basophils from the circulation to the tissues.

## Discussion

Unfortunately, the mechanism for omalizumab efficacy in urticaria is not clear (16). FcϵRI are also expressed on dendritic cells (DCs), potent antigen presenting cells, and eosinophils, and there are reports that omalizumab treatment actually deregulates FcϵRI expression on DCs (21), but it is assumed that mast cells and basophils are the main cells involved in the pathogenesis of CSU. Another evidence, as well, indicated that DCs do not play a principal role in CSU. Although a reduction in FcϵRI expression of CD1c in DCs after omalizumab treatment could be observed (10), this reduction did not correlate with clinical changes. It is well known that the expression of FcϵRI on the cell surface of mast cells is regulated by the amount of external IgE (26, 27). The same is true for basophils. This theory is supporting evidence that allergic reactions occur more quickly and severely upon a second exposure than it did after the first. The prevailing hypothesis is that omalizumab binds free IgE, which reduces FcϵRI expression on mast cells and basophils.

In atopic dermatitis patients, the omalizumab-dependent reduction of free IgE in the circulating blood is followed by the downregulation of basophilic FcϵRI expression as early as 3 days after the first dose (28). In allergic rhinitis patients, the FcϵRI expression on basophils had decreased by 88% on day 7 of omalizumab treatment, which is also when the acute allergen wheal size decreased (29). In CSU, and other allergic diseases, the mean basophil-bound IgE and basophil FcϵRI expression were noticeably reduced 7 days after initiating omalizumab treatment. These data corresponded with the onset of symptom relief, and basophilic activity remained suppressed throughout the omalizumab treatment period (30).

Our severe CSU patient responded similarly to omalizumab treatment (2). CRA1 antibody confirmed that the amount of FcϵRI expressed on basophils decreased, reflecting the result of IgE neutralization by omalizumab. CRA2 antibodies, which recognize the IgE–FcϵRI binding site, can measure unbound FcϵRI but did not detect any free FcϵRI on peripheral-blood basophils before omalizumab administration. These results mean that all the IgE receptors on peripheral-blood basophils are in a state of IgE binding. Flow cytometry confirmed that CRA2-positive basophils appeared only after external IgE was neutralized by omalizumab.

**Figure 2:**
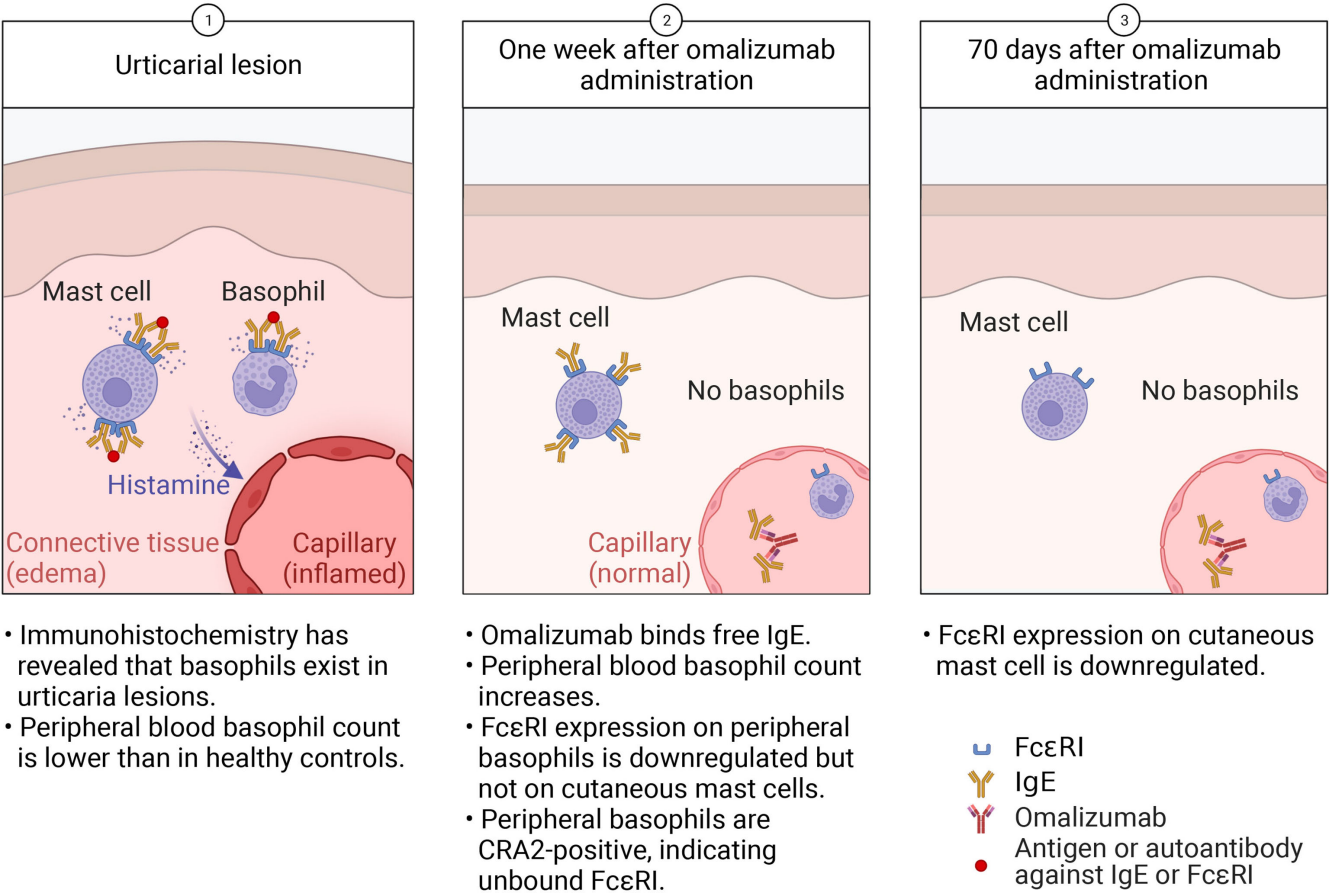
In urticaria, (Step 1) activated cutaneous mast cells and basophils release histamine, which acts on blood vessels to cause swelling. Activated basophils are recruited to the skin lesion, lowering the peripheral-blood basophil count. (Step 2) Within a week of omalizumab administration, the reduction in free IgE leads to decreased FcϵRI expression on basophils but not cutaneous mast cells. Activated, CD203c^+^ basophils stay in the peripheral blood. (Step 3) After 70 days of omalizumab treatment, mast-cell FcϵRI expression decreases but they remain in the skin.

Interestingly, the reduction of FcϵRI expression on cutaneous mast cells is much slower than for basophils (**Figure 2**). A previous report indicated that it takes 70 days after omalizumab administration for mast-cell FcϵRI expression to be significantly reduced (29). The phenotypic change in cutaneous mast cells observed with omalizumab treatment offers novel insights into the time required for tissue responses and mast cell behavior to shift in CSU (16). The same is observed in cell cultures; when human mast cells, purified from the skin are cultured *in vitro*, the FcϵRI expression does not decrease even IgE has been withdrawn for 4 to 8 weeks (31). Unlike peripheral-blood basophils, once skin mast cells have acquired FcϵRI expression, its expression does not immediately change when external IgE levels decrease. This difference may be related to the lifespan length of the two cells. In basophils, further studies are needed to determine whether the amount of IgE receptors on the same cell can change depending on the amount of external IgE, or whether new cells are mobilized from the bone marrow each time, resulting only in expressing FcϵRI dependent on the amount of external IgE.

Thus, that the early (within 7 days) onset of omalizumab’s therapeutic effects mirrors basophil dynamics and corresponds with changes in the basophil population leads us to hypothesize that basophils play a large role in CSU pathology (**Figure 2**). Although there is much to learn about urticaria, we expect that focusing on basophils will reveal new aspects of its pathogenesis.

## Author Contributions

RT-I, IK, NM, KK and NK contributed to conception and design of the study. RT-I and NK wrote the first draft of the manuscript. All authors contributed to manuscript revision, read, and approved the submitted version.

## Funding

This study was supported by a Grant-in-Aid for Young Scientists (B) to IK (Grant No. 20K17364).

## Conflict of Interest

The authors declare that the research was conducted in the absence of any commercial or financial relationships that could be construed as a potential conflict of interest.

## Publisher’s Note

All claims expressed in this article are solely those of the authors and do not necessarily represent those of their affiliated organizations, or those of the publisher, the editors and the reviewers. Any product that may be evaluated in this article, or claim that may be made by its manufacturer, is not guaranteed or endorsed by the publisher.
